# Effectiveness of remote pulmonary artery pressure estimating in heart failure: systematic review and meta-analysis

**DOI:** 10.1038/s41598-024-63742-0

**Published:** 2024-06-05

**Authors:** Szymon Urban, Oskar Szymański, Magdalena Grzesiak, Wojciech Tokarczyk, Mikołaj Błaziak, Maksym Jura, Michał Fułek, Katarzyna Fułek, Gracjan Iwanek, Piotr Gajewski, Piotr Ponikowski, Jan Biegus, Robert Zymliński

**Affiliations:** 1 Institute of Heart Diseases, University Clinical Hospital in Wroclaw, Wroclaw Medical University, Wrocław, Poland; 2 Institute of Heart Diseases, University Clinical Hospital in Wroclaw, Wrocław, Poland; 3 Department and Clinic of Internal Medicine, Occupational Diseases, Hypertension and Clinical Oncology, University Clinical Hospital in Wroclaw, Wroclaw Medical University, Wrocław, Poland; 4 Department and Clinic of Otolaryngology, Head and Neck Surgery, University Clinical Hospital in Wroclaw, Wroclaw Medical University, Wrocław, Poland; 5 Department of Physiology and Pathophysiology, Wroclaw Medical University, Wrocław, Poland

**Keywords:** Cardiology, Medical research, Heart failure

## Abstract

Heart failure (HF) poses a significant challenge, often leading to frequent hospitalizations and compromised quality of life. Continuous pulmonary artery pressure (PAP) monitoring offers a surrogate for congestion status in ambulatory HF care. This meta-analysis examines the efficacy of PAP monitoring devices (CardioMEMS and Chronicle) in preventing adverse outcomes in HF patients, addressing gaps in prior randomized controlled trials (RCTs). Five RCTs (2572 participants) were systematically reviewed. PAP monitoring significantly reduced HF-related hospitalizations (RR 0.72 [95% CI 0.6–0.87], *p* = 0.0006) and HF events (RR 0.86 [95% CI 0.75–0.99], *p* = 0.03), with no impact on all-cause or cardiovascular mortality. Subgroup analyses highlighted the significance of CardioMEMS and blinded studies. Meta-regression indicated a correlation between prolonged follow-up and increased reduction in HF hospitalizations. The risk of bias was generally high, with evidence certainty ranging from low to moderate. PAP monitoring devices exhibit promise in diminishing HF hospitalizations and events, especially in CardioMEMS and blinded studies. However, their influence on mortality remains inconclusive. Further research, considering diverse patient populations and intervention strategies with extended follow-up, is crucial for elucidating the optimal role of PAP monitoring in HF management.

## Introduction

Heart failure (HF) imposes a substantial disease burden on affected individuals. Clinical symptoms impede daily functioning, and the impact of frequent hospitalizations, which exacerbate the progression of the disease, presents a significant challenge from the patient’s perspective while also diminishing their overall quality of life^1–3^. Despite notable advancements in HF treatment modalities, encompassing pharmacological interventions and implantable devices, hospitalizations remain pervasive. It is estimated that over 50% of HF patients experience at least one hospitalization within a year^4^. Congestion, the predominant cause of HF hospitalizations, arises from intricate mechanisms regulating the interplay between volume and intravascular pressure^5,6^. Thus, although the pulmonary artery pressure does not directly represent the total body volume, its changes are interpreted as some clues about the changing congestion status of HF patients^6^.

Following this simplification and understanding some caveats of that reasoning the continuous invasive monitoring of pulmonary artery pressure (PAP) is used as a surrogate of congestion status, to assess and treat changing congestion in ambulatory HF settings. PAP, thus is an easily measurable and convenient proxy for monitoring the fluid status in HF. Importantly, an increase in PAP precedes the appearance of clinical signs of congestion in physical examinations^7^.

Previous randomized controlled trials (RCTs) using two devices, CardioMEMS^8–11^ and Chronicle^12,13^, did not provide sufficient evidence of the efficacy. While the safety of using these devices has been confirmed, their effectiveness in preventing adverse clinical outcomes is still uncertain. Given the aforementioned context, we have decided to conduct a systematic review and meta-analysis of existing data regarding the efficacy of PAP monitoring devices in the population of HF patients.

## Methods

A systematic review was conducted in adherence to the PRISMA checklist, consisting of 14 essential items^14^. The study protocol was preregistered with PROSPERO under the registration number CRD 42023449811. On the date of 12.07.2023, the initial screening and extraction of relevant papers were executed. Subsequently, two independent reviewers, namely M.G and K.F, evaluated the titles and abstracts, followed by a detailed screening of the selected full-text articles. Any disparities between the reviewers were resolved through discussion.

### Search strategy

The initial review involved the screening of the following databases:

Academic Search Ultimate, ERIC, Health Source Nursing/Academic Edition, MEDLINE, Embase, Clinicaltrials.gov, Cochrane Library (trials).

No temporal constraints were imposed on the publication timeframe.

The details of the search including keywords are available in the supplement file (File S2).

**Figure 1:**
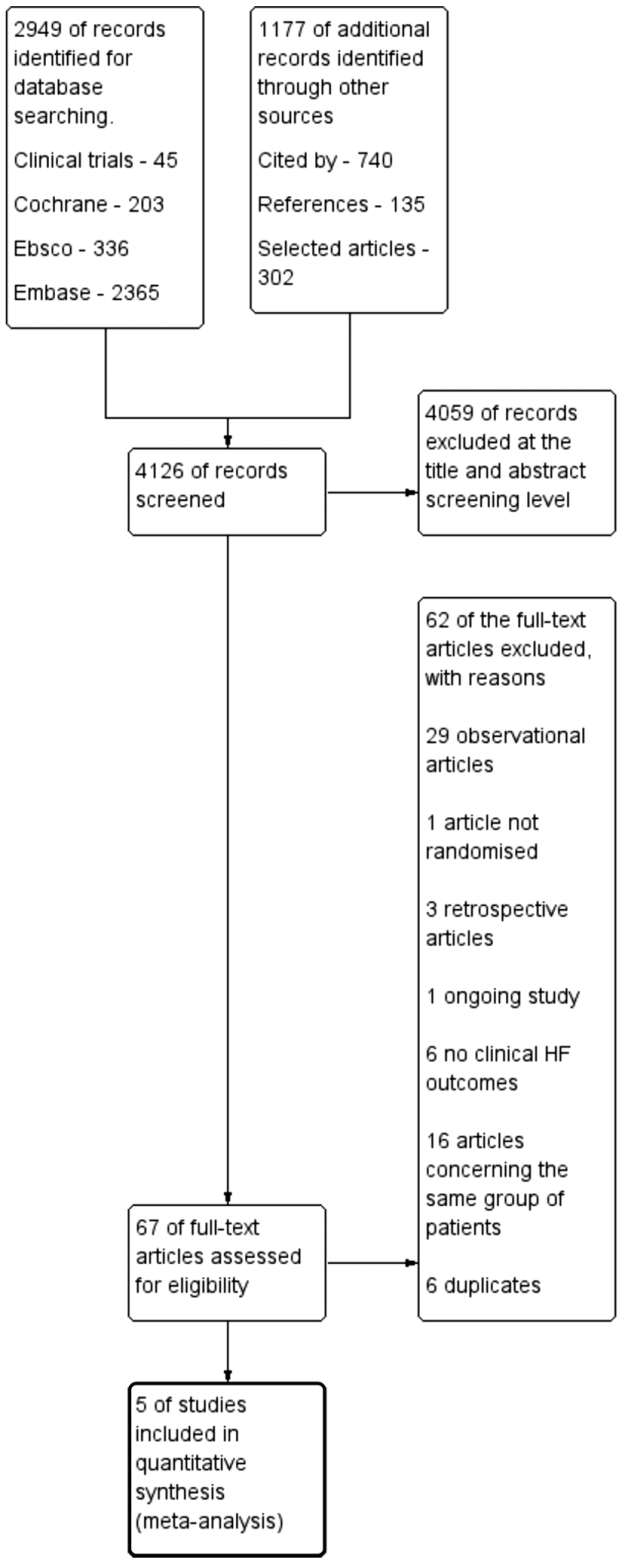
Flowchart of results of the review process.

Only studies with the status of completed, terminated, or unknown on clinicaltrials.gov were subjected to screening. Studies focusing on pediatric populations were excluded from the search. Subsequently, references and papers citing the initially included studies underwent screening for relevance. The screening process is illustrated in Fig. 1.

### Inclusion and exclusion criteria

Only double-arm, randomized controlled trials (RCT)s, analysing devices estimating the PAP were deemed eligible for inclusion in the analytical phase of this study. Specifically, peer-reviewed papers presenting full-text articles in the English language were considered for inclusion. Notably, investigations conducted on animal models or within paediatric populations were deliberately excluded from the purview of this study. Studies assessing tools measuring other hemodynamic parameters e.g. left atrium pressure, were excluded from the analysis.

### Data extraction and analysis

Two investigators, W.T and M.F, independently extracted pertinent data from the included studies, adhering to the pre-specified protocol. Preference was given to utilizing data obtained before the unmasking procedure in cases where both pre- and post-unblinding datasets were available. The extracted data is presented in Table 1.

**Table 1:** Characteristic of the included studies.

Study	Year of publication	Population	Sample size	*Intervention group, n*	*Control group, n*	*Males, %*	*Mean age, years*	Mean EF (%)	EF < 40% (%)	Implanted device	Description of intervention	Blinded
CHAMPION	2011	NYHA III	550	270	280	73	61.5	NR	78.3	CardioMems	Implantation of CARDIOMEMS and then randomization	single-blinded
GUIDE-HF	2021	NYHA II–IV	1000	497	503	63	70.5	39	49.2	CardioMems	Implantation of CARDIOMEMS and then randomization	single-blinded
MONITOR-HF	2023	NYHA III	348	176	172	76	69.5	30	71.8	CardioMems	Randomization and then implantation of CARDIOMEMS in intervention group	unmasked
REDUCEhf	2011	NYHA II-III	400	202	198	69	55	23	NR	Chronicle	Implantation of ICD and Chronicle and then randomization	single-blinded
COMPASS-HF	2008	NYHA III- IV	274	134	140	65	58	NR	NR	Chronicle	Implantation of Chronicle and then randomization	single-blinded

*EF* left ventricle ejection fraction, *NYHA* new york heart association, *NR* not reported, *HF* heart failure, *CV* cardiovascular, *I* intervention group, *C* control group, *CI* confidence interval, *PAP* pulmonary artery pressure.

For the analysis of categorical variables, Mantel–Haenszel and Inverse Variance with random effects models were employed. Odds ratios (OR), rate ratios (RR), and hazard ratios (HR) with corresponding 95% confidence intervals were calculated for various outcomes. Odds ratios were computed for events where the count of patients experiencing occurrences was compared to the total patient count, such as all-cause and cardiovascular (CV) death. In instances where the potential number of events exceeded the total patient count, as observed in HF hospitalization, rate ratios were calculated and subsequently pooled according to the guidelines outlined in the Cochrane Handbook^15^. Hazard ratios were pooled if provided in the research manuscripts. Heterogeneity was assessed using the I^2^ statistic, with I^2^ values exceeding 50% considered notable.

Funnel plots, indicative of publication bias, were not generated due to the limited number of available papers^16^. Quantitative analysis utilized Review Manager version 5.4.1 (The Cochrane Collaboration, 11–13 Cavendish Square, London, W1G 0AN United Kingdom), while figures were created using Biorender. Language editing was conducted using ChatGPT.

The authors of the papers that presented the missing data were contacted to achieve all the necessary results.

### Risk of bias and quality of evidence

The assessment of risk of bias was conducted utilizing the Cochrane Risk of Bias tool (version 2)^17^. Two independent reviewers, W.T and K.F, evaluated all included studies, and any discrepancies were resolved through discussion. A study was designated as high risk if any domain was classified as high risk or if some concerns were identified in multiple domains, substantially diminishing confidence in the study’s outcomes. Certainty of evidence underwent a similar evaluation process, employing the Grading of Recommendations Assessment, Development, and Evaluation (GRADE)^18^. GradePRO GDT (McMaster University and Evidence Prime Inc.) was employed for generating a Summary of Findings and Certainty of Evidence table.

### Subgroup, sensitivity analysis, and meta-regression

We investigated the source of heterogeneity upon its attainment of statistical significance. Subgroup analyses which assessed the outcome in CardioMEMS and Chronicle devices separately and excluded the unblinded studies (MONITOR-HF), were performed. A sensitivity analysis was performed by excluding the largest study (GUIDE-HF) to assess the durability and resilience of the results.

We conducted a meta-regression to assess the influence of the year of publication and the duration of follow-up on the intervention’s effect.

## Results

### Characteristic of the studies

The outcomes of the review process are depicted in Fig. 1. This compilation of studies involved a combined cohort of 2572 participants, distributed with 1279 individuals in the intervention group and 1293 assigned to the control group. The average age of the participants was 61.3 years, and males constituted 69% of the overall study population. Notably, three of the investigations examined the utilization of the CardioMEMS device (n = 73.79% of the entire population), while the remaining two tested the Chronicle device (n = 26.21% of the total population). The systems employ distinct methodologies for obtaining PAP. The CardioMEMS system directly measures PAP, whereas the Chronicle system assesses a range of hemodynamic parameters, including right ventricular systolic and diastolic pressures and estimated pulmonary arterial diastolic pressure (ePAD). Notably, ePAD has demonstrated a high correlation with actual PAP across various physiological conditions^19^. Except for one study exclusively involving patients with HFrEF, all other studies included patients irrespective of their ejection fraction (EF). The range of symptom severity included NYHA classes II to IV.

In four of the studies, both study groups underwent device implantation before being randomized into PAP monitoring or control groups, while one study (MONITOR-HF) exclusively performed the procedure on the intervention group. Furthermore, the research design in four of these studies maintained single-blinded conditions, ensuring that patients remained unaware of their group allocation. Conversely, one study adopted an unmasked approach. The mean duration of follow-up across these investigations was 11.25 months. The characteristic of the included studies is displayed in Table 1. In the case of the GUIDE-HF study, where data was divided into pre-covid and overall analysis, the overall data was included.

### Outcomes

**Figure 2:**
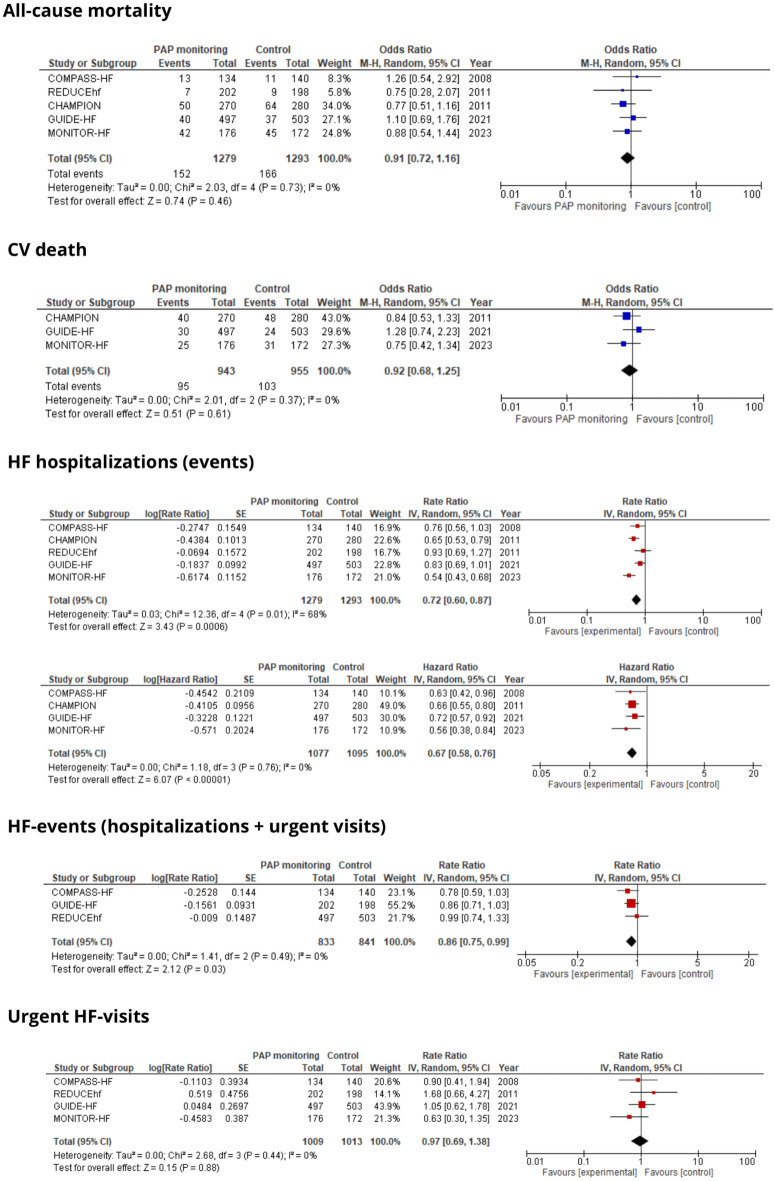
Forrest plot of the PAP monitoring versus control for the selected outcomes. *CV* cardiovascular, *HF* heart failure, *PAP* pulmonary artery pressure.

Five outcomes were consistently documented in a minimum of three studies, including all-cause and cardiovascular (CV) mortality, heart failure (HF) hospitalizations, urgent HF visits, and the composite endpoint of HF hospitalizations and urgent visits termed HF events. A quantitative analysis of these parameters was conducted, and corresponding forest plots illustrating the outcomes are presented in Fig. 2. A summary of the outcomes across the studies is provided in Table 2.

**Table 2:** Summary of findings of the included studies Number of events are displayed.

Study	Duration of follow-up, months	HF related events, n	HF hospitalisations, n	Urgent HF- visits, n	Mortality, n	CV deaths, n
CHAMPION	15	NR	I 158; C 254	NR	I 50; C 64	I 40; C 48
GUIDE-HF	12	I 213; C 252	I 185; C 225	I 28; C 27	I 40; C 37	I 30; C 24
MONITOR HF	21	NR	I 117; C 212	I 11; C 17	I 42 C 45	I 25; C 31
REDUCEhf	12	I 91; C 90	I 79; C 83	I 12; C7	I 7; C 9	NR
COMPASS	6	I 84; C 113	I 72; C 99	I 12; C14	I 13; C 11	NR

Urgent visits are defined as urgent clinic visits and emergency department visits, HF related events are definied as HF hospitalisations and urgent visits.

*HF* heart failure, *CV* cardiovascular, *I* intervention group, *C* control group.

### All-cause mortality

The all-cause mortality was reported in every study. The death occurred in the 152 (12%) patients in the study group and in the 166 (13%) in the control group. The PAP monitoring systems did not show a reduction in all-cause mortality (OR 0.91 [95% CI 0.72–1.16], *p* = 0.46). No significant heterogeneity was found.

### CV-death

Cardiovascular death was assessed in 3 studies, 95 (10%) and 103 (11%) events occurred in the study and control arm, respectively. No significant difference in the CV death was reported (OR 0.92 [95% CI 0.68–1.25], *p* = 0.61), nor relevant heterogeneity was noticed.

### HF-hospitalization

Heart failure-related hospitalization was evaluated in every study and occurred 611 (0.48 events per patient) versus 873 (0.68 events per patient) times in the study and control group respectively. The PAP monitoring reduced the HF-hospitalization rate (RR 0.72 [95% CI 0.6–0.87], *p* = 0.0006). Hazard ratio analyses, reflecting time-to-event outcomes, were conducted in four studies. The PAP monitoring reduced the risk of HF-hospitalization (HR 0.67 [95% CI 058–0.76], *p* < 0.00001).

**Figure 3:**
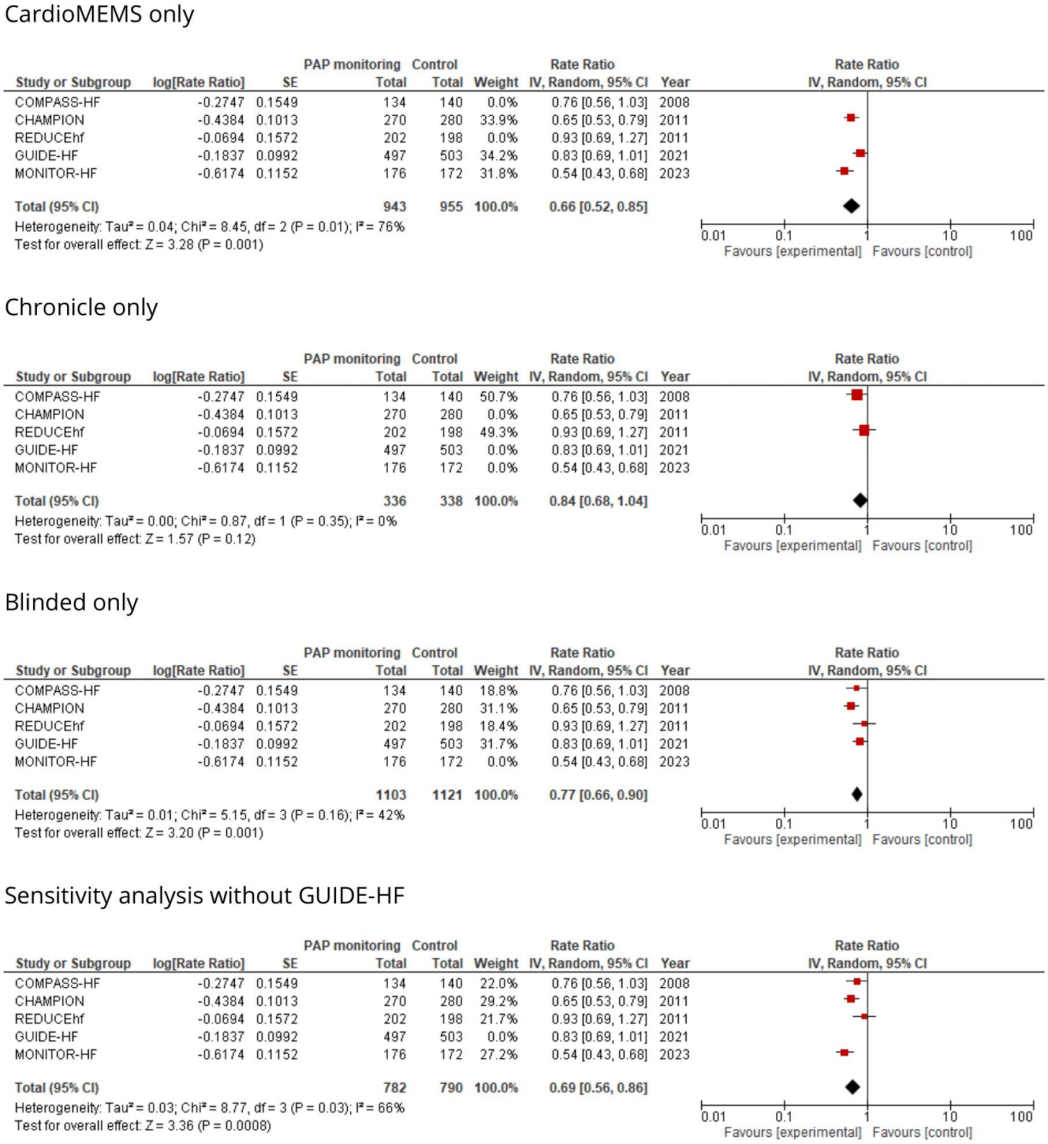
Heterogeneity analysis of the effect of PAP monitoring on HF-hospitalizations. *PAP* pulmonary artery pressure.

Significant heterogeneity was noticed in the RR analysis (I^2^ = 68%). The results retained their significance in the subgroup analysis of CardioMEMS alone (RR 0.66 [95% CI 0.52–0.85], *p* = 0.001) and in studies with a blinded design (RR 0.77 [95% CI 0.66–0.90], *p* = 0.001). However, in studies that utilized the Chronicle device, there was no significant difference between the groups (RR 0.84 [95% CI 0.68–1.04], *p* = 0.1). Furthermore, even after excluding the largest study, GUIDE-HF, for the sensitivity analysis, the findings remained statistically significant (RR 0.69 [95% CI 0.56–0.86], *p* = 0.0008). Heterogeneity analysis results are displayed in Fig. 3.

The metaregression revealed a significant association between the effect of PAP monitoring on HF hospitalization and the length of the follow-up (β = − 0.03, 95% CI = − 0.06 to 0.00, *p* = 0.039), but not with the year of the publication (β = − 0.01, 95% CI = − 0.05 to 0.02, *p* = 0.51).

### HF events (composite of the HF hospitalizations and urgent HF visits)

The HF-events were reported in 3 studies and occurred 388 (0.47 events per patient) and 441 (0.52 events per patient) times in the study and control group respectively. The PAP monitoring significantly reduced the HF events rate in the intervention group (RR 0.86 [95% CI 0.75–0.99], *p* = 0.03). No significant heterogeneity was observed.

### HF urgent visits

HF-related urgent visits were documented in four studies, with 63 events (0.06 events per patient) in the intervention group and 65 events (0.06 events per patient) in the control group. No statistical significance was observed on that field (RR 0.97 [95% CI 0.69–1.38], *p* = 0.88). No meaningful heterogeneity was observed.

### Risk of bias and certainty of evidence

**Figure 4:**
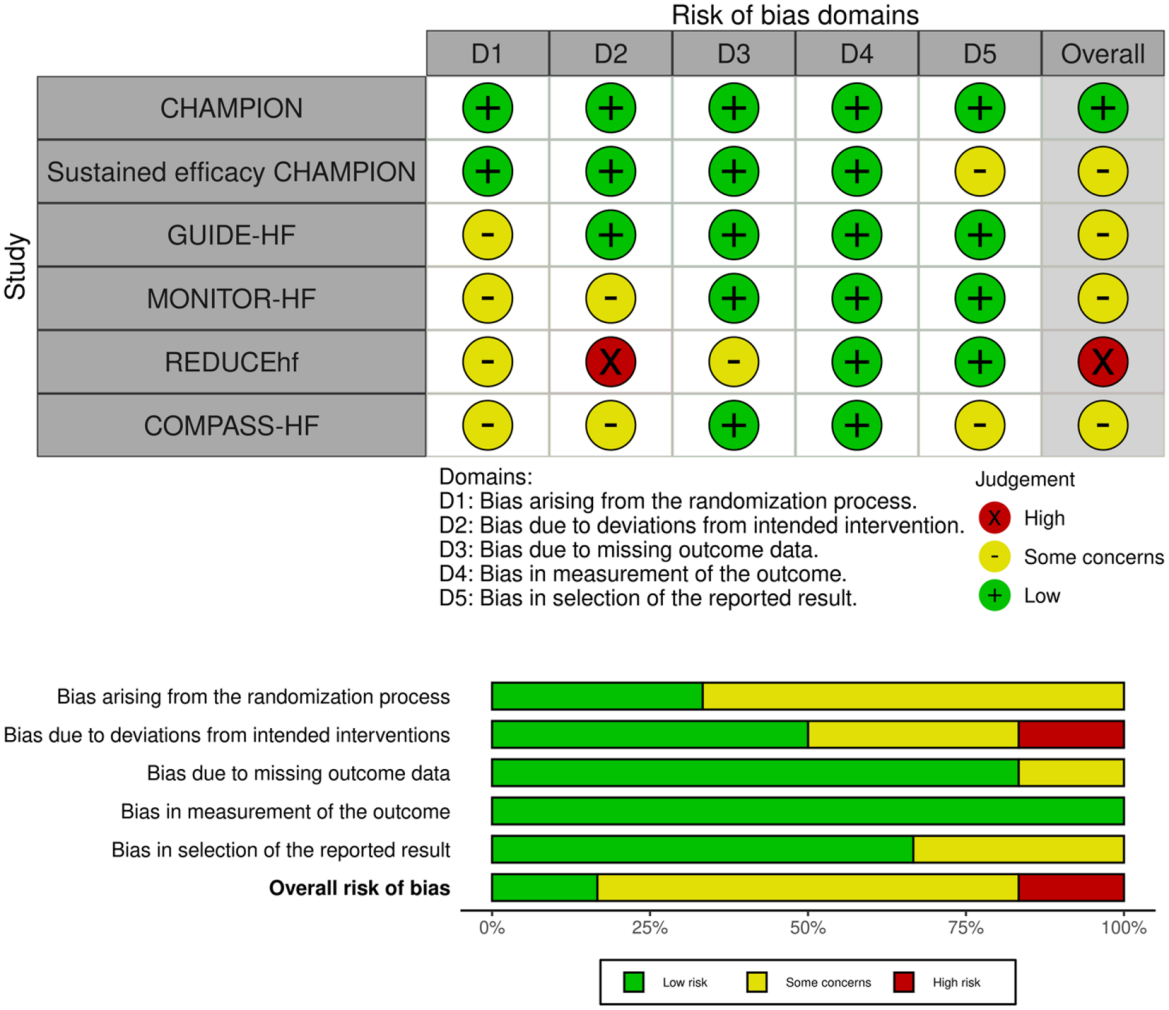
Risk of Bias in the included studies.

The results of the ROB assessment are shown in Fig. 4. The ROB was assessed as high, low or some concerns and the certainty of evidence was low or moderate depending on the outcome (Table 3).

**Table 3:** Summary of findings and certainity of evidence table.

PAP guided therapy compared to standard care for the management of HF patients					
Patient or population: chronic HF population; Setting: outpatient; Intervention: PAP guided therapy; Comparison: standard care					
Outcomes	No of participants (studies) follow-up	Certainty of the evidence (GRADE)	Relative effect (95% CI)	Anticipated absolute effects	
				Risk with standard care	Risk difference with PAP guided therapy
All cause-mortality	2572 (5 RCTs)	⨁⨁◯◯ Low^a,b,c^	OR 0.91 (0.72 to 1.16)	128 per 1000	10 fewer per 1000 (33 fewer to 18 more)
Cardiovascular death (CV death)	1898 (3 RCTs)	⨁⨁◯◯ Low^b,d^	OR 0.92 (0.68 to 1.25)	108 per 1000	8 fewer per 1000 (32 fewer to 23 more)
Heart failure hospitalizations (HF Hospitalizations)	2572 (5 RCTs)	⨁⨁◯◯ Low^a,b,e^	Rate ratio 0.72 (0.60 to 0.87)	675 per 1000	189 fewer per 1000 (270 fewer to 88 fewer)
HF-events (hospitalizations + urgent visits)	1674 (3 RCTs)	⨁⨁⨁◯ Moderate^b,f,g^	Rate ratio 0.86 (0.75 to 0.99)	541 per 1000	76 fewer per 1000 (135 fewer to 5 fewer)
Urgent HF-visits	2022 (4 RCTs)	⨁⨁◯◯ Low^a,b,h^	Rate ratio 0.97 (0.69 to 1.38)	64 per 1000	2 fewer per 1000 (20 fewer to 24 more)

The risk in the intervention group (and its 95% confidence interval) is based on the assumed risk in the comparison group and the relative effect of the intervention (and its 95% CI). *CI* confidence interval; *OR* odds ratio. Explanations: High certainty: we are very confident that the true effect lies close to that of the estimate of the effect. Moderate certainty: we are moderately confident in the effect estimate: the true effect is likely to be close to the estimate of the effect, but there is a possibility that it is substantially different. Low certainty: our confidence in the effect estimate is limited: the true effect may be substantially different from the estimate of the effect. Very low certainty: we have very little confidence in the effect estimate: the true effect is likely to be substantially different from the estimate of effect.

^a^The majority of studies were rated with some concerns in the randomization process domain, two studies were rated with some concerns in the selection of the reported results domain and deviations from the intended interventions domain, and one study was rated High risk of bias in deviations from intended intervention domain.

^b^All studies report direct outcomes in the HF patients population irrespective of LVEF in NYHA spectrum II–IV, comparing PAP monitoring versus standard care.

^c^The total number of events does not meet the optimal size. 95% CI includes no effect and appreciable benefit however the sample size is very large.

^d^The total number of events does not meet the optimal size. 95% CI includes no effect and appreciable benefit and harm.

^e^Substantial heterogeneity cannot be fully explained.

^f^All studies have been rated some concerns in the randomization process domain, one study was rated the high risk of bias in deviations from the intended intervention domain.

^g^The total number of events meets the optimal size of 400, by the rule of thumb, to meet the threshold. 95% CI includes no effect but does not include appreciable benefit or harm. h. The total number of events does not meet the optimal size. 95% CI includes no effect and appreciable benefit and harm, however the sample size is very large.

## Discussion

In our meta-analysis, we have evaluated the value of continuous remote PAP monitoring in the HF population. The data comes from, the 5 RCTs analysing 2 different devices measuring the PAP. The principal findings of our analysis indicate that treatment based on these devices exhibited a statistically significant reduction in HF-related hospitalizations and HF events, albeit without a significant impact on overall mortality. Notably, this association remained statistically significant in the aggregate analysis of data from the CardioMEMS device, but not in the case of the Chronicle device. Importantly, this significance persisted even after we excluded the largest study within our dataset, (the GUIDE-HF trial). Furthermore, our findings were consistent regardless of the publication date of the studies and revealed a noteworthy association between the duration of follow-up and the extent of reduction in heart failure (HF) hospitalizations within the device arm. Specifically, studies with a more extended observation period demonstrated a more pronounced reduction in HF-related hospitalizations. This meta-analysis represents the most comprehensive examination to date, comprising a substantial cohort of 2572 participants. It encompasses a comprehensive review of all existing data regarding PAP estimating devices in the context of HF populations, drawing exclusively from RCTs.

The design of the included studies varied considerably. Notably, MONITOR-HF was the sole unblinded study, potentially influencing outcomes, particularly self-reported measures such as quality of life^20^. The impact of the COVID-19 pandemic on the outcomes of PAP monitoring warrants further exploration. Notably, the GUIDE HF trial has published analyses both pre-pandemic and during the pandemic. Enrollment for this trial concluded in 2019, and by the onset of the pandemic lockdown, 72% of the follow-up was complete, with 42% of patients having finished. Pre-pandemic analyses indicated positive outcomes, which lost significance during the pandemic. This change was primarily due to a reduction in events, especially heart failure-related hospitalizations and visits, in the control group, with no significant changes in the intervention arm. The pandemic also led to a decrease in cardiovascular events across multiple RCTs^21^. This reduction in heart failure-related hospitalizations could be attributed to patients’ reluctance to seek hospital care due to fear of COVID-19 infection and a greater focus on health maintenance, potentially improving adherence^22^.

The recent meta-analysis regarding the topic was published simultaneously with the last RCT—MONITOR HF^23^. The analysis evaluated the effect of the CardioMEMs devices on the selected outcomes and revealed a significant reduction of the: HF hospitalizations, the composite of HF hospitalizations, urgent visits and all-cause mortality, composite of HF hospitalizations and all-cause mortality and composite of HF-hospitalization and urgent HF visits. No significant interactions in the analysed subgroups were found, and no metaregression was performed.

The similar meta-analysis also analysed pooled results of studies regarding CardioMEMS and Chronicle device^24^. Laconelli et al evaluated the results of CHAMPION, COMPASS-HF, GUIDE-HF, and REDUCEhf trials. The use of PAP monitoring devices significantly reduced HF-hospitalizations, and did not affect all-cause mortality. No data regarding the subgroups or meta-regression were published. Our results complement the previous data with the information coming from the studies analysing all the available devices for PAP monitoring. The differences in the number of included studies stem from the fact that, compared to the study by Clephas et al., our meta-analysis, except the results from the CardioMEMs trials, incorporates results from studies evaluating the effectiveness of the Chronicle system. Additionally, unlike the analysis by Laconelli et al., our work also includes results from the MONITOR HF study, which was not available at the time of their analysis. In summary, the data from PAP monitoring and therapeutic interventions translate into a reduction in the risk of HF hospitalization. Interestingly, this does not appear to correlate with a reduction in mortality risk, which may stem from several factors. Firstly, it is challenging to expect that monitoring alone would reduce any endpoint because it merely provides information. The crucial factor is what actions the physician takes based on this information. So, if the primary intervention aimed at reducing PAP is an increase in diuretic dosage, as was the case in the included studies, it may not translate into a better prognosis, as we know that diuretics do not themselves improve prognosis but rather alleviate clinical symptoms of congestion. As demonstrated in the STRONG-HF study, the optimization of neurohormonal blockade proves to be a safe strategy, irrespective of the patient’s age, comorbidities, or natriuretic peptide level^25–28^. Therefore, the development of a response algorithm to elevated PAP based not only on the escalation of the diuretics but rather, on the optimization of the guideline-directed medical therapy (GDMT) i.e. renin–angiotensin–aldosterone system (RAAS) blockade and sodium–glucose cotransporter 2 (SGLT-2) inhibitors, both of which possess decongestion-promoting properties, including indirect reductions in PAP, might yield more favourable outcomes^29–31^. Furthermore, the absence of a positive effect on mortality may be attributed to the characteristics of the population included in the analysed trials, wherein a substantial proportion consisted of patients with HFpEF. Given that individuals with HFpEF frequently experience non-cardiovascular mortality^32^, demonstrating a mortality reduction with a device in this particular population proves challenging.

Although the results of PAP monitoring remained consistent across subgroups in the analysis by Clephas et al., the device appears to yield more favourable outcomes in the most vulnerable patient populations, such as elderly patients with NYHA class III symptoms, which aligns with intuition, as this group would inherently have a higher baseline hospitalization rate. Ultimately, the most appropriate and potentially beneficial patient population for PAP monitoring remains to be determined, and further research is needed to clarify its role in clinical practice. Subgroup analysis of specific populations in studies involving the Chronicle device was not conducted due to the absence of reported data. As a result, any additional subgroup analyses would have been limited to data from the CardioMEMS, thus duplicating the analysis previously published by Clephas et al. Our distinctive contributions, apart from the exclusive analysis of the Chronicle device, include analyses confined to blinded studies and a sensitivity analysis that excludes the largest study (Fig. 3). Notably, the positive effect persisted as significant in both analyses, which reinforces the reliability of our findings.

Novel devices designed for monitoring PAP are presently under scrutiny^33^, with one particularly promising solution being the Cordella. Apart from assessing hemodynamic parameters, the device acquires and transmits the vitals. Notably, the Cordella stands out as the inaugural device that permits and encourages patients to self-monitor PAP and autonomously adjust therapy. Prior endeavors involving the incorporation of patients into self-management based on hemodynamic parameters, as conducted in the LAPTOP-HF trial, yielded promising results^34^. Preliminary findings from the SIRONA-2 trial, focused on assessing the safety and efficacy of the Cordella device, exhibit a favorable outlook^35–37^.

There is a possibility that optimizing the results could be achieved by extending the duration of follow-up. Our findings suggest that the favourable impact of PAP monitoring may become more prominent with longer observation periods. Therefore, it is advisable to conduct further studies that include a more carefully selected patient population, potentially those at a higher risk of hospitalizations, extend the pharmacological intervention beyond the diuretic escalation, and prolong the blinded observation period. Such investigations are warranted to gain a deeper understanding of the benefits of PAP monitoring.

Notably, in the largest study (GUIDE-HF), the control group received bi-weekly calls from healthcare providers, and the outcomes did not show significant differences between the groups. Patients in the control group, often receive less frequent follow-up, which may explain the difference in events. Importantly, there is substantial evidence supporting the effectiveness of non-invasive telemonitoring systems for heart failure (HF) patients, which have demonstrated their efficacy in reducing more durable outcomes, including all-cause mortality^38^. Conversely, the landscape of telemonitoring methods remains heterogeneous, marked by instances of inefficacious telemonitoring approaches^39–42^. Consequently, additional trials are imperative to systematically assess and compare the impact of invasive hemodynamic monitoring against specifically chosen non-invasive telemonitoring systems, which have demonstrated proven efficacy.

## Limitations

Our study is not devoid of limitations. Firstly, we were restricted to utilizing only aggregated data from the trials, and conducting a patient-level analysis could potentially provide more in-depth and nuanced conclusions. It is worth noting that all the trials were conducted in the United States and the Netherlands, which are both highly developed countries with relatively robust healthcare systems. The trails were not powered to detect the differences in mortality. Furthermore, it is important to acknowledge that the physicians were not blinded to the allocation of patients, which inherently introduces a vulnerability to performance bias, The risk of bias remains significant, even though it is challenging to conceive a study design in which the physician would not have access to data from the implanted sensor^43^. Finally, the Chronicle device is presently obsolete and lacks FDA approval. Additionally, it does not directly measure the PAP but provides estimates. This meta-analysis sought to assess the impact of remote PAP measurement-driven therapy on outcomes, focusing on the method rather than the specific device.

In conclusion, our meta-analysis contributes additional evidence regarding the potential efficacy of PAP monitoring devices in reducing HF hospitalizations. However, it is imperative to emphasize that the clinical impact of this procedure warrants further comprehensive evaluation and scrutiny. Further research is needed to better understand the true clinical significance and optimal application of PAP monitoring devices in the management of heart failure.

### Supplementary Information

Supplementary Information.

## Data Availability

The datasets uses and/or analysed during the current study available from the corresponding author on reasonable request.
