# Supplementary material for: Effectiveness of remote pulmonary artery pressure estimating in heart failure: systematic review and meta-analysis
Source: Sci Rep. 2024 Jun 5;14:12929. doi: 10.1038/s41598-024-63742-0 (PMC11153505; doi:10.1038/s41598-024-63742-0)
Supplement: Supplementary file 1 — Supplementary Information. [file 41598_2024_63742_MOESM1_ESM.docx]

**The details of the search:**

The keyword used in specific databases differed slightly. In EMBASE, Academic Search Ultimate, ERIC, Health Source Nursing/Academic Edition and Cochrane Library, keywords were as follows: (pulmonary AND artery) AND (monitoring) AND ((heart failure) OR (ventricular dysfunction) OR (HF) OR (HFpEF) OR (HfrEF) OR (cardiomyopat*) OR (((cardia*) OR (myocardial)) AND ((failure) OR (insufficienc*)))).

Searching in Clinicaltrials.gov included the following conditions: Condition or disease: ((heart failure) OR (ventricular dysfunction) OR (HF) OR (HfpEF) OR (HfrEF) OR (cardiomyopat*) OR (((cardia*) OR (myocardial)) AND ((failure) OR (insufficienc*)))) Other terms: (pulmonary AND artery) AND (monitoring).
